# Uniform intratumoral distribution of radioactivity produced using two different radioagents, ^64^Cu-cyclam-RAFT-c(-RGDfK-)_4_ and ^64^Cu-ATSM, improves therapeutic efficacy in a small animal tumor model

**DOI:** 10.1186/s13550-018-0407-3

**Published:** 2018-06-19

**Authors:** Zhao-Hui Jin, Atsushi B. Tsuji, Mélissa Degardin, Aya Sugyo, Yukie Yoshii, Kotaro Nagatsu, Ming-Rong Zhang, Yasuhisa Fujibayashi, Pascal Dumy, Didier Boturyn, Tatsuya Higashi

**Affiliations:** 1 National Institute of Radiological Sciences, National Institutes for Quantum and Radiological Science and Technology, Anagawa 4-9-1, Inage, Chiba, 263-8555 Japan; 2 Département de Chimie Moléculaire-UMR CNRS 5250, Université Grenoble Alpes, 38041 Grenoble Cedex 9, France; 3 IBMM, UMR-5247, Université de Montpellier, CNRS, École Nationale Supérieure de Chimie de Montpellier, 34296 Montpellier Cedex 5, France

**Keywords:** Intratumoral radioactivity distribution, Combination targeted radionuclide therapy, Tumor co-targeting strategy, Therapy response evaluation, Radiopharmaceuticals, ^64^Cu-labeled multimeric cRGD peptide, ^64^Cu-ATSM, α_V_β_3_ integrin, Angiogenesis, Hypoxia

## Abstract

**Background:**

The present study proposed a new concept for targeted radionuclide therapy (TRT) to improve the intratumoral distribution of radioactivity using two different radiopharmaceuticals. We examined the efficacy of a combination of a tetrameric cyclic Arg-Gly-Asp (cRGD) peptide-based radiopharmaceutical, ^64^Cu-cyclam-RAFT-c(-RGDfK-)_4_ (^64^Cu-RaftRGD, an α_V_β_3_ integrin [α_V_β_3_] tracer), and ^64^Cu-diacetyl-bis (*N*^4^-methylthiosemicarbazone) (^64^Cu-ATSM, a supposed tracer for hypoxic metabolism) in a small animal tumor model.

**Results:**

Mice with subcutaneous α_V_β_3_-positive U87MG glioblastoma xenografts were used. The intratumoral distribution of a near-infrared dye, Cy5.5-labeled RAFT-c(-RGDfK-)_4_ (Cy5.5-RaftRGD), ^64^Cu-RaftRGD, and ^64^Cu-ATSM was visualized by fluorescence imaging and autoradiography of the co-injected Cy5.5-RaftRGD with ^64^Cu-RaftRGD or ^64^Cu-ATSM at 3 h postinjection. Mice were treated with a single intravenous dose of the vehicle solution (control), 18.5 or 37 MBq of ^64^Cu-RaftRGD or ^64^Cu-ATSM, or a combination (18.5 MBq of each agent). The tumor volume, tumor cell proliferation, body weight, survival, and tumor and organ uptake of radiopharmaceuticals were assessed. It was shown that Cy5.5-RaftRGD colocalized with ^64^Cu-RaftRGD and could be used as a surrogate for the radioactive agent. The intratumoral distribution of Cy5.5-RaftRGD and ^64^Cu-ATSM was discordant and nearly complementary, indicating a more uniform distribution of radioactivity achievable with the combined use of ^64^Cu-RaftRGD and ^64^Cu-ATSM. Neither ^64^Cu-RaftRGD nor ^64^Cu-ATSM showed significant effects on tumor growth at 18.5 MBq. The combination of both (18.5 MBq each) showed sustained inhibitory effects against tumor growth and tumor cell proliferation and prolonged the survival of the mice, compared to that by either single agent at 37 MBq. Interestingly, the uptake of the combination by the tumor was higher than that of ^64^Cu-RaftRGD alone, but lower than that of ^64^Cu-ATSM alone. The kidneys showed the highest uptake of ^64^Cu-RaftRGD, whereas the liver exhibited the highest uptake of ^64^Cu-ATSM. No obvious adverse effects were observed in all treated mice.

**Conclusions:**

The combination of ^64^Cu-RaftRGD and ^64^Cu-ATSM achieved an improved antitumor effect owing to the more uniform intratumoral distribution of radioactivity. Thus, combining different radiopharmaceuticals to improve the intratumoral distribution would be a promising concept for more effective and safer TRT.

**Electronic supplementary material:**

The online version of this article (10.1186/s13550-018-0407-3) contains supplementary material, which is available to authorized users.

## Background

Targeted radionuclide therapy (TRT) is a promising cancer treatment modality, which is implemented by systemic administration of a specific targeting vehicle, such as an antibody, peptide, and small organic molecule, that is radiolabeled with an α- or β^−^-particle emitter or Auger electron emitter to deliver a cytocidal radiation dose to both the primary and metastatic lesions [1]. However, the intrinsically heterogeneous biological properties of tumors would lead to heterogeneous intratumoral distribution of the TRT agent. Thus, delivering sufficient radiation throughout the entire tumor to achieve a greater treatment efficacy remains challenging. If we could find a therapeutic option that could achieve homogeneous intratumoral distribution of radionuclides, this might improve the therapeutic efficacy. There are many types of TRT agents that can target various biological properties; combining different agents may achieve a homogeneous distribution of radionuclides, resulting in delivery of sufficient radiation throughout the entire tumor.

^64^Cu is a promising theranostic radionuclide owing to its suitable half-life (12.7 h) with multiple decay modes, including β^+^ (18%), which is used for positron emission tomography (PET) imaging, β^−^ (39%, 0.95−1.4 mm tissue range) [2], and Auger electron (43%, ~ 126 nm tissue range) [3], which are used for therapeutic radiation. We previously developed a tetrameric cyclic Arg-Gly-Asp (cRGD) peptide-based radiopharmaceutical, ^64^Cu-cyclam-RAFT-c(-RGDfK-)_4_ (^64^Cu-RaftRGD) [4] for targeting α_V_β_3_ integrin (α_V_β_3_), a transmembrane glycoprotein receptor that is highly expressed on both the angiogenic endothelial and tumor cells and plays important roles in tumor growth, invasion, metastasis, and angiogenesis [5, 6]. Our previous studies showed that ^64^Cu-RaftRGD PET could detect α_V_β_3_-positive tumors and angiogenesis, as well as monitor the antiangiogenic effects [4, 7, 8]; in addition, we showed the therapeutic efficacy of ^64^Cu-RaftRGD as an α_V_β_3_-targeted radionuclide therapy [9]. Moreover, we focused on another Cu(II)-based theranostic agent, ^64^Cu-diacetyl-bis (*N*^4^-methylthiosemicarbazone) (^64^Cu-ATSM), which supposedly accumulates in regions of hypoxic metabolism within the tumor [10, 11]. As a neutral lipophilic small molecule with high membrane permeability and low redox potential, Cu-ATSM was designed to release Cu(I) ions within cells under over-reduced conditions (such as hypoxia) but not under normal conditions, leading to selective intracellular trapping of Cu(I) ions [12]. Clinical PET studies showed the association between the tumor uptake of ^60//62/64^Cu-ATSM and therapeutic resistance, metastatic potential, and poor prognosis [10, 11]; in addition, preclinical studies using animal tumor models showed the antineoplastic activity of ^64^Cu-ATSM [13–15].

Considering that ^64^Cu-RaftRGD and ^64^Cu-ATSM most likely accumulate in different regions of the tumor, with the former accumulating in regions of angiogenesis (toward a normoxic state), and the latter accumulating in regions of hypoxic metabolism, and that angiogenesis and hypoxic metabolism may co-exist since both are common biological features of solid malignancy [16, 17], we hypothesized that a more uniform radioactivity distribution could be achieved via co-injection of the two agents into the tumor. Herein, we aimed to address a new concept for TRT to improve the intratumoral radioactivity distribution using two different radiopharmaceuticals, by studying the combined use of ^64^Cu-RaftRGD and ^64^Cu-ATSM in an α_V_β_3_-positive tumor mouse model.

## Methods

### Radiopharmaceuticals and peptide probe

^64^Cu was produced at the National Institute of Radiological Sciences facility (Chiba, Japan) with a molar activity of 1.77 ± 1.17 TBq/μmol and a radionuclidic purity of > 99.9% [18]. ^64^Cu labeling of cyclam-RAFT-c(-RGDfK-)_4_ and H_2_ATSM was performed, as previously described [4, 19] (brief description in Additional file 1). ^64^Cu-RaftRGD was produced with a molar activity of 148 MBq/nmol and radiochemical purity of > 99.5%. The radiochemical purity of ^64^Cu-ATSM was > 95%. A near-infrared dye, Cy5.5-conjugated RAFT-c(-RGDfK-)_4_ (Cy5.5-RaftRGD) was also prepared, as previously described [20].

The two radioagents were mixed at a radioactivity ratio of 1:1, kept at room temperature, and analyzed using a reversed-phase high-performance liquid chromatography system, as described in Additional file 1 for determination of the co-administration mode (simultaneously or sequentially).

As previously reported [9, 21], for in vivo use, ^64^Cu-RaftRGD was formulated in a vehicle solution of normal saline containing 1% Tween 80 to improve the peptide solubility, Gelofusine® (80 mg/kg, Braun Medical, Oss, Netherlands), and l-lysine (400 mg/kg, Sigma-Aldrich, Buchs, Switzerland) to reduce the renal retention of the tracer (by > 50%). ^64^Cu-ATSM was diluted with normal saline only [22].

### Animals and tumor model

Animal procedures were approved by the Animal Ethics Committee of the National Institutes for Quantum and Radiological Science and Technology (Chiba, Japan) and were conducted in accordance with the institutional guidelines. The α_V_β_3_-positive tumor model was induced by subcutaneous inoculation of 5 × 10^6^ U87MG human glioblastoma cells (American type culture collection) into the right flank of female BALB/cAJcl-*nu/nu* mice (5 weeks old, CLEA Japan, Inc.), as previously reported [9]. The size of the tumor mass was measured with a digital caliper, tumor volume (mm^3^) was determined using the following formula: tumor volume = 0.5 × length × width^2^, and the tumor growth ratio was calculated by dividing the obtained value by the initial tumor volume measured immediately before treatment (day 0). Unless otherwise stated, the mice that had a median body weight value of 21 g (range, 19−23 g) and developed tumors of approximately 100 mm^3^ at 14 days post-tumor cell inoculation were used for subsequent studies. The humane survival endpoint of mice was determined when the tumor size exceeded 1500 mm^3^ [9]. It should be emphasized that all animal experiments performed in the present study including those presented in additional files were designed to use the same strain, sex, and age of mice obtained from the same company as abovementioned, and the tumor-bearing mice were randomly divided into groups for subsequent studies.

### Intratumoral distribution of radiopharmaceuticals

Mice were pretreated (via the tail vein) with 37 MBq (0.25 nmol) of ^64^Cu-RaftRGD, and 10 min later, 20 nmol of Cy5.5-RaftRGD was injected (to avoid competitive blocking). Another group of mice bearing tumors of various sizes were co-injected with 37 MBq of ^64^Cu-ATSM and 20 nmol of Cy5.5-RaftRGD. At 3 h postinjection (p.i.), the mice were euthanized and tumors were excised. Autoradiography, fluorescence microscopic examination, and hematoxylin and eosin (HE) staining were sequentially performed using the same tumor frozen sections. In addition, autoradiography, immunofluorescence staining of the microvascular marker, CD31, and HE staining were performed using adjacent sections. See Additional file 1 for the detailed procedures. The staining for α_V_β_3_ and a hypoxia marker was omitted because previous studies have shown the colocalization of α_V_β_3_ staining and ^64^Cu-RaftRGD distribution in the tumor model used here [9] and the correlation of ^64^Cu-ATSM uptake with the expression of hypoxia-inducible factor-1α (a protein of cellular response to hypoxia) in human tumors [23] and xenografts [24].

### Therapeutic experiments

Mice (*n* = 5−6/group) were treated with a single administration of the vehicle solution (control), 18.5 or 37 MBq of ^64^Cu-RaftRGD (0.125 and 0.25 nmol for 18.5 and 37 MBq, respectively) or ^64^Cu-ATSM (2.5 and 5 nmol for 18.5 and 37 MBq, respectively), or a combination of both at a dose of 18.5 MBq for each agent (radioactivity ratio = 1:1, for the simplicity of study design). Then, the body weight of mice and tumor size were measured every 2 days, and the survival endpoint days (as defined above) were recorded.

In a separate experiment, mice (*n* = 4/group) were treated with 111 or 148 MBq of the combination to see if the use of higher doses (enhancing the radiation energy) can cause more effective antitumor effect (inducing tumor shrinkage compared to the tumor size before treatment). Hematology and hepatorenal function tests were carried out for toxicity evaluation, as described in Additional file 1.

### Biodistribution study

Mice (*n* = 5/group) were treated with 0.74 MBq of ^64^Cu-RaftRGD (0.25 nmol), ^64^Cu-ATSM (5 nmol), or the combination (0.125 nmol of ^64^Cu-RaftRGD plus 2.5 nmol of ^64^Cu-ATSM). The “nmol” amounts indicated above were the values adjusted to those used for the 37 MBq therapy protocols. At 3 and 24 h p.i., the mice were euthanized, and the blood, tumor, muscles, liver, and kidneys were collected and measured using a gamma counter with decay correction. Tumor and organ uptake was expressed as the percentage of injected radioactivity per gram of tissue (%IA/g) normalized to 20 g body weight by multiplying with the ratio of body weight to 20 g.

### Histological evaluation of tumor proliferation

Mice (*n* = 3−4/group at each time point) were injected with the vehicle solution or 37 MBq of ^64^Cu-RaftRGD, ^64^Cu-ATSM, or the combination. At 1 and 3 days p.i., the mice were euthanized and tumors were excised for immunohistochemical staining of the proliferation marker, Ki67. As previously described [9], the proliferation index was defined as the percentage of Ki67-positive cells, which was quantified by counting five areas (× 40 objective lens) of highest Ki67 staining intensity using the WinROOF 2015 image analysis software (version: 3.10.0, Mitani Corporation, Fukui, Japan). See Additional file 1 for the detailed procedures.

### Statistical analysis

Quantitative data were presented as the means ± standard deviations. Unpaired *t* test and one-way analysis of variance followed by Dunnett’s test were used for two-group and multiple comparisons, respectively (Kaleida Graph 4.0). Based on the period from day 0 p.i. to the specified survival endpoint day, Kaplan-Meier survival curves were plotted, and the differences in survival were evaluated using the log-rank test (GraphPad Prism 5). *P* values < 0.05 were considered statistically significant.

## Results

### Radiopharmaceuticals

**Fig. 1:**
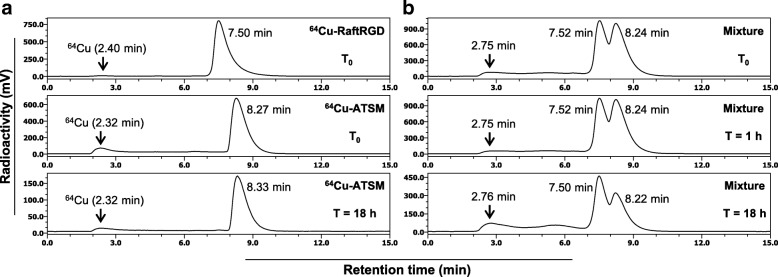
Representative chromatograms of **a** ^64^Cu-RaftRGD and ^64^Cu-ATSM labeling mixture immediately after the radiolabeling reaction (*T*_0_) or after 18 h of storage at room temperature (*T* = 18 h), and **b** the mixture of ^64^Cu-RaftRGD and ^64^Cu-ATSM immediately after mixing (*T*_0_) or after 1 and 18 h of storage at room temperature (*T* = 1, 18 h)

^64^Cu-ATSM could be analyzed using the chromatography system established for ^64^Cu-RaftRGD analysis (Fig. 1a). The same method was used for analysis of ^64^Cu-RaftRGD and ^64^Cu-ATSM mixed at equal amounts of radioactivity. The chromatograms obtained immediately after mixing and after 1-h storage at room temperature showed two nearly identical radioactivity peaks corresponding to ^64^Cu-RaftRGD and ^64^Cu-ATSM (Fig. 1b). The obtained chromatogram after overnight (18 h) incubation showed a decrease in the radioactivity peak of ^64^Cu-ATSM, compared to that of ^64^Cu-RaftRGD (Fig. 1b), whereas no obvious change was detected for ^64^Cu-ATSM alone at the same time (Fig. 1a). These results showed the high stability of both ^64^Cu-RaftRGD and ^64^Cu-ATSM in their mixture for at least 1 h, indicating that the two radioagents were co-administrable without obvious drug-drug interactions within 1 h after mixing the two agents.

### Intratumoral distribution of ^64^Cu-RaftRGD and ^64^Cu-ATSM

**Fig. 2:**
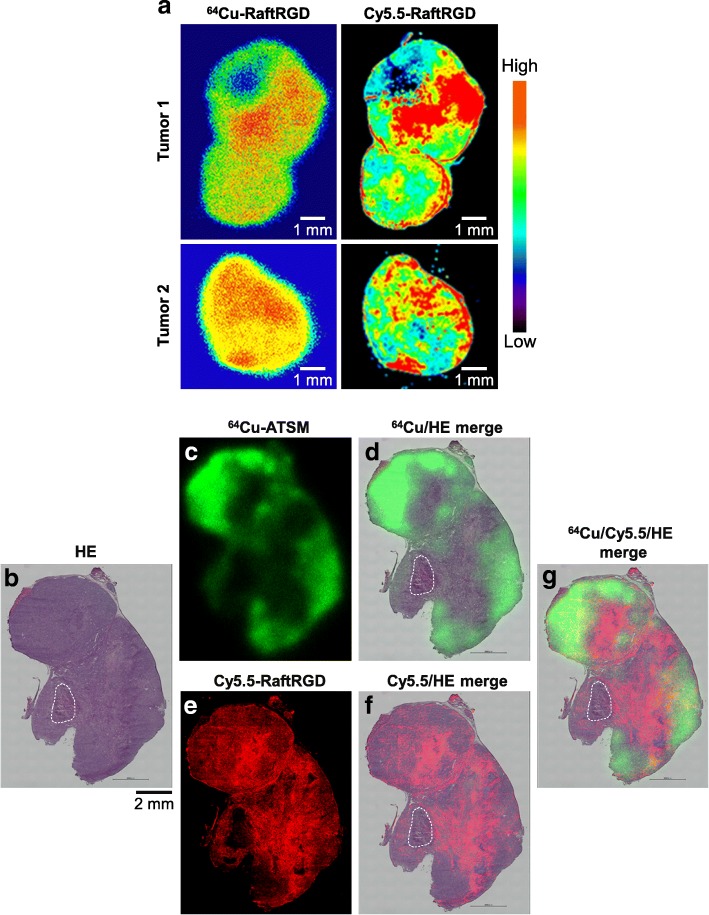
**a** Well-matched intratumoral distribution of ^64^Cu-RaftRGD and Cy5.5-RaftRGD. Mice bearing U87MG tumors were pre-injected with 37 MBq of ^64^Cu-RaftRGD, injected 10 min later with 20 nmol of Cy5.5-RaftRGD, and euthanized at 3 h p.i. Autoradiography and fluorescence imaging were sequentially performed in the same tumor section. Left panels, ^64^Cu autoradiograms; right panels, Cy5.5 fluorescence acquired using the Odyssey CLx near-infrared fluorescence imaging system. **b**−**g** A representative tumor section showing distribution of ^64^Cu-ATSM and Cy5.5-RaftRGD. U87MG tumor-bearing mice were co-injected with ^64^Cu-ATSM and Cy5.5-RaftRGD and euthanized at 3 h p.i. Autoradiography, fluorescence imaging, and HE staining were sequentially performed in the same tumor section. **b** HE staining; **c** ^64^Cu autoradiogram in green; **d** Merged image of **b** and **c**; **e** Cy5.5 fluorescence in red; **f** Merged image of **b** and **e**; **g** Merged image of **b**, **c**, and **e**. Yellow, red/green overlay. The necrotic region is marked by a dotted line. Scale bar, 2 mm

Figure 2a shows well-matched distribution patterns of ^64^Cu-RaftRGD and Cy5.5-RaftRGD in two tumor sections. This finding validated the feasibility of the use of Cy5.5-RaftRGD, as a representative of ^64^Cu-RaftRGD, to discriminate the spatial distribution of ^64^Cu-RaftRGD from that of ^64^Cu-ATSM in the same tumor. Figure 2b–g shows a series of images acquired on the same tumor section; ^64^Cu-ATSM autoradiograph (green), fluorescence image of Cy5.5-RaftRGD (red), and HE micrograph. The “green” HE (Fig. 2d) and “red” HE (Fig. 2f) merged images show discordant distribution of ^64^Cu-ATSM and Cy5.5-RaftRGD. Higher levels of ^64^Cu-ATSM radioactivity were observed in the outer regions of the tumor, whereas Cy5.5-RaftRGD accumulated mainly in the inner regions. The “green,” “red,” and HE merged images (Fig. 2g) further suggested the complementary distribution of ^64^Cu-ATSM and Cy5.5-RaftRGD, despite limited overlaps (yellow). Except for the necrotic area, Cy5.5-RaftRGD generally accumulated in the regions showing low ^64^Cu-ATSM accumulation and vice versa. Similar results were also found in the other five tumors of various sizes (50−1065 mm^3^, see Additional file 2). An autoradiographed slice adjacent to the one shown in Fig. 2b was further examined by CD31 and HE staining. As shown in Additional file 3, CD31-stained microvessels were extensively observed in both the high and low ^64^Cu-ATSM regions; however, their size was generally smaller in the high ^64^Cu-ATSM regions than in the low ^64^Cu-ATSM regions.

### Radiotherapy with ^64^Cu-RaftRGD, ^64^Cu-ATSM, and a combination of both

**Fig. 3:**
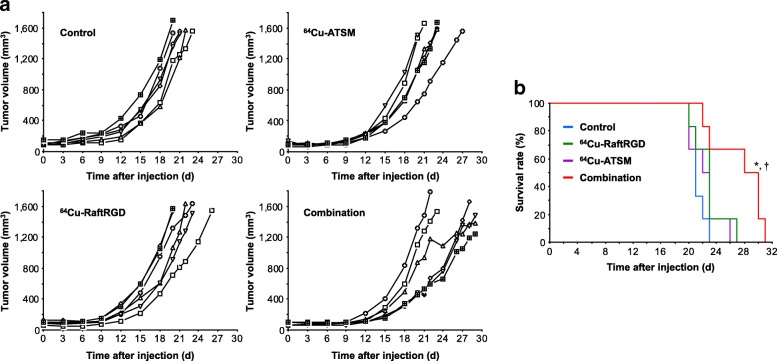
Tumor growth curves (**a**) and Kaplan-Meier endpoint-survival curves (**b**) of U87MG tumor-bearing mice after single injection of the vehicle solution (control) or 37 MBq of ^64^Cu-RaftRGD, ^64^Cu-ATSM, or a combination (18.5 MBq for each agent). *n* = 6/group; ^*^P < 0.01 and ^†^*P* < 0.05 for the combination group vs. control group and vs. ^64^Cu-RaftRGD or ^64^Cu-ATSM group, respectively

Neither ^64^Cu-RaftRGD nor ^64^Cu-ATSM showed significant effects on the tumor growth at 18.5 MBq (Additional file 4). Figure 3a and Additional file 5a shows the tumor growth curves of mice treated with the same total radioactivity dose (37 MBq) of ^64^Cu-RaftRGD, ^64^Cu-ATSM, or a combination (18.5 MBq of each agent). No significant difference was found in the initial tumor volumes among the groups. From day 3 to 9 p.i., all treatments at 37 MBq resulted in a significant delay of tumor growth, compared to the vehicle-treated control: for example, on day 9, the tumor volumes were 117 ± 33 mm^3^ (*P* = 0.0018), 126 ± 20 mm^3^ (*P* = 0.006), and 87 ± 13 mm^3^ (*P* < 0.0001) for ^64^Cu-RaftRGD, ^64^Cu-ATSM, and combination groups, respectively, vs. 189 ± 46 mm^3^ for the control group. From day 12 to 20, only the combination maintained the significant tumor growth-delaying effects: for example, on day 12, the tumor volumes were 233 ± 78 mm^3^ (*P* > 0.05), 208 ± 26 mm^3^ (*P* > 0.05), and 129 ± 42 mm^3^ (*P* = 0.004) for ^64^Cu-RaftRGD, ^64^Cu-ATSM, and combination groups, respectively, vs. 275 ± 102 mm^3^ for the control group. Furthermore, the endpoint survival analysis (Fig. 3b) showed that the median survival days of the control, ^64^Cu-RaftRGD, ^64^Cu-ATSM, and combination groups were 21, 22.5, 23, and 29, respectively. The combination group exhibited significantly prolonged survival, compared to the control, ^64^Cu-RaftRGD, and ^64^Cu-ATSM groups. Changes in body weight were also compared among groups (Additional file 5b). The mice treated with either ^64^Cu-RaftRGD or ^64^Cu-ATSM alone showed slightly but significantly lower body weights on day 3, compared to the control mice, whereas no significant body weight loss was observed for the combination group.

In comparison with the 37 MBq combination (Additional file 6a), increasing the total radioactivity dose of the combination to 111−148 MBq induced significant tumor shrinkage in a dose-dependent manner (Additional file 6b); additionally, it did not cause a marked or long-lasting body weight loss (Additional file 6c). The 148 MBq-treated mice showed a maximal mean body weight reduction of 9.9% (less than 10%) on day 6, compared to day 0 (baseline), and afterwards the body weight gradually recovered beyond the baseline. Measurement of hematology and hepatorenal functions (Additional file 7) did not show a dose-response relationship between the 111 and 148 MBq doses. All the values obtained on day 21 after treatment were not significantly different from those measured in the control mice.

### Histological study of tumor proliferation

**Fig. 4:**
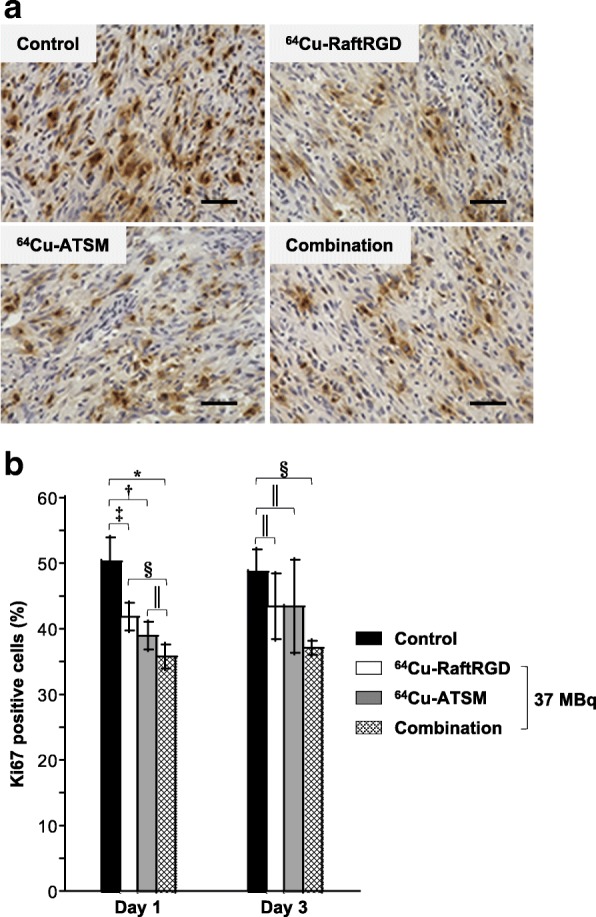
Ki67 staining (dark brown) of the tumor sections at days 1 and 3 p.i. of the vehicle solution (control) or 37 MBq of ^64^Cu-RaftRGD, ^64^Cu-ATSM, or a combination (18.5 MBq for each agent). **a** Representative images at day 1. Scale bar, 50 μm. Nuclei were lightly stained with hematoxylin (blue). **b** Quantitative analysis of Ki67 staining (the percentage of positively stained cells). *n* = 3−4/group; ^*, †, ‡, §^*P* < 0.0001, 0.001, 0.01, and 0.05, respectively; ^║^*P* > 0.05

Results of the histological analysis of cell proliferation, evaluated by Ki67 staining, in the treated and control tumors are presented in Fig. 4. On day 1 p.i. of ^64^Cu-RaftRGD, ^64^Cu-ATSM, or the combination at 37 MBq, all treated tumors showed a significant reduction of the proliferation index (the percentage of Ki67-positive cells), compared to the vehicle-treated control. The proliferation index of the combination-treated tumors was generally smaller than that of either single agent-treated tumors; a statistically significant difference was observed between the combination and ^64^Cu-RaftRGD groups; however, the difference between the combination and ^64^Cu-ATSM groups did not reach statistical significance. On day 3 p.i., only the combination-treated tumors continued to exhibit a significantly reduced proliferation index.

### Biodistribution of ^64^Cu-RaftRGD, ^64^Cu-ATSM, and a combination of both

The biodistribution data obtained at 3 and 24 h p.i. of ^64^Cu-RaftRGD (0.74 MBq), ^64^Cu-ATSM (0.74 MBq), or a combination (0.37 MBq of each agent) are summarized in Table 1. Data represented the tumor and organ uptake levels from the therapeutic dose (37 MBq) because the same peptide or ATSM dose was used. ^64^Cu-RaftRGD and ^64^Cu-ATSM exhibited rapid blood clearance and low levels of activity in the muscles. The highest radioactivity accumulation was found in the kidneys followed by the liver for ^64^Cu-RaftRGD, and in the liver followed by the kidneys for ^64^Cu-ATSM. The tumor uptake levels of ^64^Cu-ATSM were higher than those of ^64^Cu-RaftRGD in the U87MG tumor model. The two agents showed similar biodistribution kinetics. From 3 to 24 h p.i., the blood, liver, kidney, muscle, and tumor uptake of ^64^Cu-RaftRGD decreased by 20, 33, 29, 40, and 23%, respectively. Nearly similar results were observed for ^64^Cu-ATSM (20, 39, 17, 14, and 39%, respectively). The tumor and organ uptake values of the combination were very close to the average of the values of the two single agents. Tumor uptake at 3 and 24 h was 1.6- and 1.4-fold higher for the combination (10.7 ± 1.05 and 7.02 ± 1.20 %IA/g) than ^64^Cu-RaftRGD (6.69 ± 0.64 and 5.18 ± 0.24 %IA/g), respectively. However, it was 1.4- and 1.3-fold lower than that of ^64^Cu-ATSM (15.5 ± 2.03 and 9.47 ± 0.78 %IA/g, respectively).

**Table 1:** Biodistribution of ^64^Cu-RaftRGD, ^64^Cu-ATSM, and a combination of both in U87MG tumor-bearing mice

Organ	3 h p.i.				24 h p.i.			
	^64^Cu-RaftRGD (a)	^64^Cu-ATSM (b)	Combination	(“a” + “b”)/2	^64^Cu-RaftRGD (a)	^64^Cu-ATSM (b)	Combination	(“a” + “b”)/2
Blood	0.05 ± 0.01	2.1 ± 0.12	1.15 ± 0.14^*a, *b^	1.08	0.04 ± 0.01	1.68 ± 0.15	0.83 ± 0.06^*a, *b^	0.86
Liver	8.06 ± 0.56	19.9 ± 1.41	12.8 ± 0.42^*a, *b^	14	5.43 ± 0.27	12.1 ± 0.69	8.48 ± 0.28^*a, *b^	8.77
Kidneys	11.8 ± 0.86	8.38 ± 0.49	9.55 ± 0.79^†a, §b^	10.1	8.42 ± 0.61	6.98 ± 0.34	7.18 ± 0.38^‡a,║b^	7.7
Muscles	0.4 ± 0.06	0.73 ± 0.06	0.57 ± 0.06^†a, ‡b^	0.57	0.24 ± 0.04	0.63 ± 0.05	0.46 ± 0.06^*a, †b^	0.44
Tumor	6.69 ± 0.64	15.5 ± 2.03	10.7 ± 1.05^‡a, †b^	11	5.18 ± 0.24	9.47 ± 0.78	7.02 ± 1.2^‡a, ‡b^	7.33

Tissue radioactivity was assessed at 3 and 24 h p.i. and expressed as %IA/g. *n* = 5/group; ^*a, †**a**, ‡**a**^*P* < 0.0001, 0.001, and 0.01, respectively vs. the ^64^Cu-RaftRGD group; ^*b, †**b**, ‡**b**, §**b**^*P* < 0.0001, 0.001, 0.01, and 0.05, respectively or ^**║b**^*P* > 0.05 vs. the ^64^Cu-ATSM group

## Discussion

To the best of our knowledge, the present proof-of-concept study is the first to report the use of a combination of different radiopharmaceuticals to improve the heterogeneous intratumoral distribution of radionuclides and enhance the therapeutic efficacy. So far, the most commonly used strategy to improve the efficacy of TRT involves its combination with other treatment modalities, such as chemotherapy and external radiotherapy [25].

It is of great importance to determine a suitable co-administration mode (simultaneous or sequential) for combination of different drugs owing to the possibility of in vitro and in vivo drug-drug interactions, as well as the difference in biopharmacokinetics of the drugs. In the present study, we selected the simultaneous mode based on the results of the chromatographic analysis of ^64^Cu-RaftRGD and ^64^Cu-ATSM mixture, which did not indicate any radioagent-radioagent reaction, such as transchelation (owing to the potentially different ^64^Cu-coordinating ability of the two agents) within 1 h after mixing the two agents. In addition, our previous data did not show different biodistribution patterns of the two radiopharmaceuticals [21, 22]. In addition, the results of the present biodistribution study using the same experimental settings further showed no radioagent-radioagent reactions in vivo, as evidenced by the fact that the measured tumor and organ uptake values for the co-injected ^64^Cu-RaftRGD and ^64^Cu-ATSM were approximately equal to the average values of those obtained for the single agents.

Expectedly, the intratumoral distribution study showed spatially discordant but complementary distribution patterns of ^64^Cu-RaftRGD and ^64^Cu-ATSM in the α_V_β_3_-positive U87MG tumor xenograft, which resulted in a more uniform radioactivity distribution in the tumors co-injected with the two agents. Immunostaining with CD31 revealed that the regions of high ^64^Cu-ATSM and low ^64^Cu-RaftRGD accumulation were enriched with relatively smaller microvessels than the regions of low ^64^Cu-ATSM and high ^64^Cu-RaftRGD accumulation. This difference in the microvasculature indicated the complementary relationship between ^64^Cu-ATSM and ^64^Cu-RaftRGD accumulation according to the intratumoral vascularization levels, which is in accordance with our proof of concept in the present study.

In the subsequent therapeutic study, despite using the same total radioactivity dose (37 MBq), the combined use of ^64^Cu-RaftRGD and ^64^Cu-ATSM (18.5 MBq of each agent) was more efficient in maintaining its inhibitory effects against tumor growth and tumor cell proliferation and prolonging the survival of mice than either single agent. At a dose of 18.5 MBq, neither ^64^Cu-RaftRGD nor ^64^Cu-ATSM showed significant effects on tumor growth. Hence, these results might indicate a synergistic antitumor effect between ^64^Cu-RaftRGD and ^64^Cu-ATSM. We further examined the total tumor radioactivity uptake of ^64^Cu-RaftRGD, ^64^Cu-ATSM, and the combination at the therapeutic dose. Lower tumor uptake values were observed for the more effective combination therapy (10.7 ± 1.05 and 7.02 ± 1.20 %IA/g at 3 and 24 h, respectively), compared to the corresponding values of ^64^Cu-ATSM alone (15.5 ± 2.03 and 9.47 ± 0.78 %IA/g, respectively), indicating no decisive correlation between the tumor uptake and antitumor effects. Taken together, results of the intratumoral distribution, total tumor uptake levels, and therapeutic effects suggested that the improved antitumor effects of the combination therapy could be largely attributed to the improved intratumoral radioactivity distribution. Regarding the therapeutic efficacy of ^64^Cu-RaftRGD plus ^64^Cu-ATSM combination, a dose-response relationship was revealed; a total dose of 37 MBq induced tumor growth delay, whereas higher doses (111 and 148 MBq) caused tumor growth inhibition. It should be mentioned that to simplify the experiment schedules, the mice were treated with only one single-dose injection, but despite this, significant tumor growth delay and promising results of tumor growth inhibition were achieved by the combination with the total doses of 37 MBq and 111−148 MBq, respectively. Actually, in clinical settings, TRT as well as other treatment modalities such as chemotherapy and external radiotherapy are administered or performed multiple times to obtain the most effective therapeutic efficacy. Thus, it may be anticipated that multiple-dose regimen of the combined TRT with ^64^Cu-RaftRGD and ^64^Cu-ATSM could greatly improve the treatment effectiveness.

For TRT, the presence of dose-limiting organs usually restricts the administered activity levels of therapeutic radiopharmaceuticals. Radiopharmaceuticals considered for combined use should have different critical organ profiles, which may lower the risk of dose-limiting toxicity and widen the therapeutic window. Previous studies showed that the kidneys constitute the principal dose-limiting organ for ^64^Cu-RaftRGD therapy [9], whereas the liver, red marrow, and ovaries are the dose-limiting organs for ^64^Cu-ATSM therapy [22]. In the present study, the kidneys exhibited the highest tissue uptake of ^64^Cu-RaftRGD followed by the liver, whereas the liver showed the highest tissue uptake of ^64^Cu-ATSM followed by the kidneys. Throughout the study (up to 3−4 weeks after treatment), we did not observe any serious side effects even in the mice treated with the highest dose (148 MBq; 74 MBq of ^64^Cu-RaftRGD plus 74 MBq of ^64^Cu-ATSM). Our previous study showed that ^64^Cu-ATSM at 148 MBq caused hematological toxicity, which might be related to the resultant poor survival of the treated mice [15]. Although further long-term evaluation of safety is needed, the combination therapy has the potential to be safer, compared to dose escalation of a single agent.

So far, only few studies have reported the combined use of different radiopharmaceuticals for TRT [26]. Previous studies performed TRT of neuroendocrine tumors via the combined use of high-energy and long-path ^90^Y-somatostatin analog with its medium energy and short path ^177^Lu analog for combating tumors of various sizes [27], or with ^131^I-metaiodobenzylguanidine (a norepinephrine analog) for increasing the dose delivered to the tumor site [28]. Different from their treatment concepts, the present study suggested the combination of different radiopharmaceuticals (may not be limited to the currently used agents) to improve the intratumoral radioactivity distribution and thus enhance the therapeutic efficacy. Despite different concepts, all combined TRT treatments might have a common advantage: allowance of personalized and optimized dose calculation for each radiopharmaceutical based on the dosimetric data and spatial distribution in the tumor obtained from separate PET scans because many TRT agents or their surrogates are also PET tracers.

The performance of the proposed newly designed treatment concept should be evaluated in further studies using various tumor models and, if possible, different combinations of radioagents with optimized doses for each single agent to maximize the therapeutic efficacy. Another limitation of the present study is the lack of information on the radiation absorbed dose distribution in tumors, which might be more directly related to the tumor response. However, to obtain these data, a sophisticated three-dimensional dose calculation technique would be needed [29].

## Conclusions

In conclusion, the combined use of two different radioagents, ^64^Cu-RaftRGD and ^64^Cu-ATSM with discordant intratumoral distribution patterns resulted in a more uniform intratumoral radioactivity distribution, resulting in an improved antitumor effect. Combining different radiopharmaceuticals to improve the intratumoral distribution would be a promising concept for more effective and safer TRT.

## Additional files

Additional file 1: Supplementary materials and methods. (PDF 95 kb)

Additional file 2: Intratumoral distribution of ^64^Cu-ATSM and Cy5.5-RaftRGD. Mice bearing U87MG tumors with sizes of 50–1065 mm^3^ were co-injected with ^64^Cu-ATSM and Cy5.5-RaftRGD and euthanized 3 h later. Autoradiography, fluorescence imaging and HE staining were sequentially performed in the same tumor sections. Merged images showing ^64^Cu autoradiogram in green, Cy5.5 fluorescence in red, and HE stains. Yellow, red/green overlay. The necrotic regions are surrounded by dotted lines. Scale bars, 1, 2 mm. (PDF 235 kb)

Additional file 3: Intratumoral distribution of ^64^Cu-ATSM and microvasculature. An adjacent slice to that presented in Fig. 2b was examined by autoradiography, CD31 immunofluorescence staining, and HE staining. Merged image showing ^64^Cu-ATSM distribution in green, CD31-stained microvessels in red, and HE stains. High-resolution pictures shown by dotted rectangles clearly depicting the stained microvessels in smaller size in ^64^Cu-ATSM high- vs. low-accumulated areas. Nuclei were stained with DAPI (blue). Scale bars, 2 mm, 200 μm. (PDF 285 kb)

Additional file 4: Tumor growth curves of U87MG tumor-bearing mice after single injection of the vehicle solution (control) or 18.5 MBq of ^64^Cu-RaftRGD (**a**) or ^64^Cu-ATSM (**b**). *n* = 5–6/group. (PDF 116 kb)

Additional file 5: Tumor growth curves (**a**) and body weight changes (**b**) of the same set of experimental groups as described in Fig. 3. Values are the means ± standard deviations (*n* = 6/group). The final data points shown for each group of mice (**b**) represent the results obtained at survival endpoint days (represented by a mean value). ^*, †, ‡^*P* < 0.05 for combination, ^64^Cu-RaftRGD, and ^64^Cu-ATSM vs. vehicle control, respectively. (PDF 140 kb)

Additional file 6: (**a**) Tumor growth ratios of the same set of treated groups as described in Fig. 3. ^*, †, ‡^*P* < 0.05 for combination, ^64^Cu-RaftRGD, and ^64^Cu-ATSM vs. vehicle control, respectively. Tumor growth ratios (**b**) and body weight changes (**c**) of U87MG tumor-bearing mice after co-administration of ^64^Cu-RaftRGD and ^64^Cu-ATSM at 111 MBq (55.5 MBq for each agent) and 148 MBq (74 MBq for each agent). Values are the means ± standard deviations (*n* = 4/group). ^*,^^**^P < 0.05 and 0.01, respectively for 111 MBq-group vs. 148 MBq group, respectively. It should be noted that although vehicle controls (**b**, **c**) were not performed simultaneously along with the 111 MBq and 148 MBq groups, all the three independent experiments (**#**1 and **#**2 extracted from Additional file 4 and Additional file 6a, respectively) showed a reproducibly steady increase of the tumor volume in the vehicle-treated mice. (PDF 337 kb)

Additional file 7: Hematology (**a**) and hepatorenal functions (**b**) of U87MG tumor-bearing mice after co-administration of ^64^Cu-RaftRGD and ^64^Cu-ATSM at 111 or 148 MBq. (PDF 100 kb)

## Abbreviations

α_V_β_3_: α_V_β_3_ integrin
%IA/g: Percentage of injected radioactivity per gram of tissue
^64^Cu-ATSM: ^64^Cu-diacetyl-bis (*N*^4^-methylthiosemicarbazone)
^64^Cu-RaftRGD: ^64^Cu-cyclam-RAFT-c(-RGDfK-)_4_
cRGD: Cyclic Arg-Gly-Asp
Cy5.5-RaftRGD: Cy5.5-labeled RAFT-c(-RGDfK-)_4_
HE: Hematoxylin and eosin
p.i.: Postinjection
PET: Positron emission tomography
TRT: Targeted radionuclide therapy
