# Supplementary material for: Uniform intratumoral distribution of radioactivity produced using two different radioagents, 64Cu-cyclam-RAFT-c(-RGDfK-)4 and 64Cu-ATSM, improves therapeutic efficacy in a small animal tumor model
Source: EJNMMI Res. 2018 Jun 19;8:54. doi: 10.1186/s13550-018-0407-3 (PMC6008272; doi:10.1186/s13550-018-0407-3)
Supplement: Supplementary file 1 — Supplementary materials and methods. (PDF 95 kb) [file 13550_2018_407_MOESM1_ESM.pdf]

Additional file 1: Supplementary materials and methods

Preparation of ^{64}Cu-cyclam-RAFT-c(-RGDfK-)$_4$ (^{64}Cu-RaftRGD) and ^{64}Cu-diacetyl-bis (*N*^{4}-methylthiosemicarbazone) (^{64}Cu-ATSM)

For preparation of ^{64}Cu-RaftRGD, 1 nmol of cyclam-RAFT-c(-RGDfK-)$_4$ (molecular weight: 4119.6) in 20 μL of dimethyl sulfoxide was mixed with 148 MBq of $^{64}CuCl_2$ in 20 μL of ammonium citrate buffer (100 mM, pH 5.5) and incubated at 70 °C for 10 min. The radiolabeling efficiency was assessed by reversed-phase high-performance liquid chromatography (solvent A, 0.1% trifluoroacetic acid in H_2O; solvent B, 0.1% trifluoroacetic acid in acetonitrile; flow rate = 1.3 mL/min, linear gradient, 5 to 100% solvent B in 15 min; Cosmosil 5C18-MS-II column, 4.6 ID × 150 mm [Nacalai Tesque, Inc., Kyoto, Japan]). The analysis was performed on a Waters chromatography system (Nihon Waters K.K., Tokyo, Japan) equipped with a Waters 1525 binary pump, Waters 2489 dual absorbance detector, and radiation detection system (Ludlum Model 44-10 γ-scintillator and Model 2200 Scaler/Ratemeter, Ludlum Measurements, Inc., Sweetwater, Texas). The relative radioactivity was expressed in millivolts (mV).

For preparation of ^{64}Cu-ATSM, 1 nmol of H_2ATSM (molecular weight: 261.37) in 1 μL of dimethyl sulfoxide was mixed with 7.4 MBq of $^{64}CuCl_2$ in 1 μL of ammonium citrate buffer and incubated at room temperature for 15 min. The radiolabeling efficiency was determined by silica gel thin-layer chromatography (silica gel 60; Merck, Darmstadt, Germany) using ethyl acetate as a mobile phase.

Radioactivity levels on the thin-layer chromatography plates were analyzed using a bioimaging analyzer (FLA-7000; Fujifilm, Tokyo, Japan).

Autoradiography and fluorescence imaging

The excised tumors were embedded in Tissue-Tek OCT compound (Sakura Finetek, Tokyo, Japan), and frozen by immersion in *n*-hexane precooled at −80 °C. Frozen sections (10 µm thick) were then made, air-dried, and kept in the dark. For autoradiography, after overnight exposure of the tumor sections to an imaging plate (BAS-MS 2040, Fujifilm) at −80 °C, the plate was scanned using a bioimaging analyzer (FLA-7000) for determination of intratumoral radioactivity distribution. The sections were then stored at −80 °C until the radioactivity decayed to negligible levels, after which the sections were fixed with 2% paraformaldehyde at room temperature for 10 min, mounted with mounting agent (Dapi-Fluoromount-G^{TM}; SouthernBiotech, Birmingham, AL) containing 4′,6-diamidino-2-phenylindole for nucleus staining, and observed for intratumoral Cy5.5 fluorescence distribution. An adjacent autoradiographed section was fixed in cold acetone at −20 ºC for 10 min, stained with a rat anti-mouse CD31 monoclonal antibody (1:1500 dilution; BD Biosciences, Bedford, MA), and visualized using Alexa Fluor 594-conjugated goat anti-rat antibody (1:200 dilution; Invitrogen, Camarillo, CA). After autoradiography and fluorescence imaging, the slides were immersed in phosphate-buffered saline at 4 ºC for a couple of days to remove the coverslips, and then stained with hematoxylin and eosin (HE). Fluorescence and HE images of the whole-

tumor sections were acquired using a fluorescence microscope (BZ-9000, Keyence, Osaka, Japan) or the Odyssey CLx near-infrared fluorescence imaging system (LI-COR Biotechnology, Lincoln, NE) as indicated.

Histological study of tumor proliferation

The excised tumors were embedded in Tissue-Tek OCT compound and frozen by immersion in *n*-hexane precooled at −80 °C. Frozen sections (10 μm thick) were cut and stored at −80 °C until the radioactivity decayed to negligible levels. The sections were fixed with 4% paraformaldehyde at room temperature for 15 min, incubated with a rabbit anti-human Ki67 antibody (SP6, 1:500 dilution; Abcam, Cambridge, UK) followed by a peroxidase-labeled polymer-conjugated goat anti-rabbit immunoglobulin (Dako, Glostrup, Denmark), and visualized with the chromogen diaminobenzidine. Nuclear counterstaining was carried out with hematoxylin.

Hematology and hepatorenal functions

Examination of the hematological and hepatorenal functions was performed as previously described (1). For hematology, 10 μL of tail vein blood was examined in a hematology analyzer (Celltac α MEK-6458, Nihon Kohden, Tokyo, Japan) for measurement of white blood cell count (WBC, 10^2/μL), red blood cell count (RBC, 10^4/μL), platelet count (PLT, 10^4/μL), hemoglobin concentration (HGB, g/dL), hematocrit value (HCT, %), and red blood cell indices, including mean

cell volume (MCV, fL), mean cell hemoglobin (MCH, pg), and mean cell hemoglobin concentration (MCHC, g/dL). For hepatorenal function test, the blood collected by cardiac puncture was examined in a blood chemistry analyzer (FDC7000V, Fujifilm) for determination of the levels of blood urea nitrogen (BUN, mg/dL), creatinine (CRE, mg/dL), glutamate oxaloacetate transaminase (GOT, U/L), glutamate pyruvate transaminase (GPT, U/L), gamma-glutamyl transpeptidase (GGT, U/L), and alkaline phosphatase (ALP, U/L).

Supplementary reference

1. Jin ZH, Furukawa T, Degardin M, et al. $\alpha v\beta_3$ Integrin-targeted radionuclide therapy with ^{64}Cu-cyclam-RAFT-c(-RGDfK-)$_4$. Mol Cancer Ther. 2016;15(9):2076–85.
