# Supplementary material for: Uniform intratumoral distribution of radioactivity produced using two different radioagents, 64Cu-cyclam-RAFT-c(-RGDfK-)4 and 64Cu-ATSM, improves therapeutic efficacy in a small animal tumor model
Source: EJNMMI Res. 2018 Jun 19;8:54. doi: 10.1186/s13550-018-0407-3 (PMC6008272; doi:10.1186/s13550-018-0407-3)
Supplement: Supplementary file 2 — Intratumoral distribution of 64Cu-ATSM and Cy5.5-RaftRGD. Mice bearing U87MG tumors with sizes of 50–1065 mm3 were co-injected with 64Cu-ATSM and Cy5.5-RaftRGD and euthanized 3 h later. Autoradiography, fluorescence imaging and HE staining were sequentially performed in the same tumor sections. Merged images showing 64Cu autoradiogram in green, Cy5.5 fluorescence in red, and HE stains. Yellow, red/green overlay. The necrotic regions are surrounded by dotted lines. Scale bars, 1, 2 mm. (PDF 235 kb) [file 13550_2018_407_MOESM2_ESM.pdf]

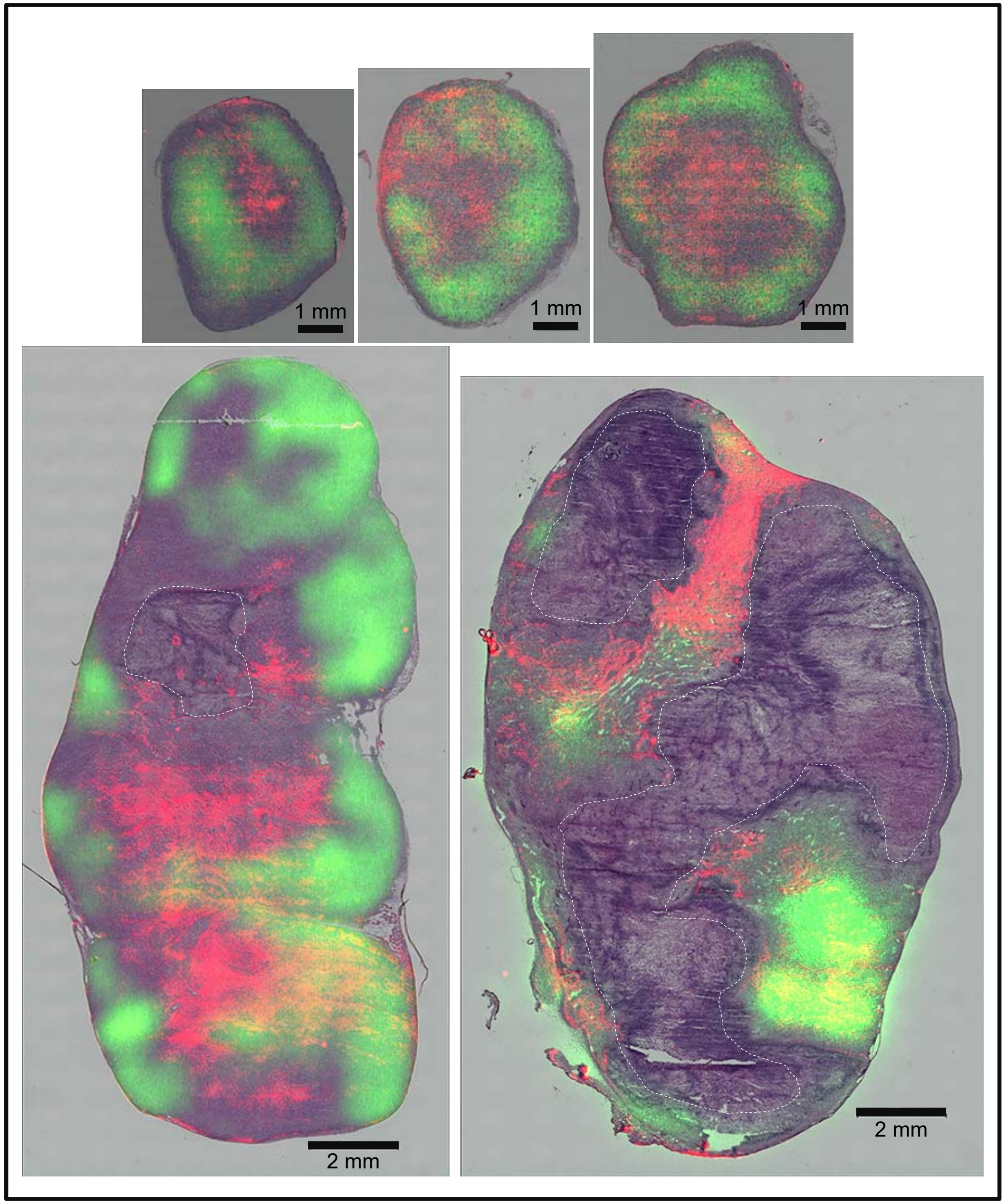

Additional file 2. Intratumoral distribution of ^{64}Cu-ATSM and Cy5.5-RaftRGD. Mice bearing U87MG tumors with sizes of 50–1065 mm^3 were co-injected with ^{64}Cu-ATSM and Cy5.5-RaftRGD and euthanized 3 h later. Autoradiography, fluorescence imaging and HE staining were sequentially performed in the same tumor sections. Merged images showing ^{64}Cu autoradiogram in green, Cy5.5 fluorescence in red, and HE stains. Yellow, red/green overlay. The necrotic regions are surrounded by dotted lines. Scale bars, 1, 2 mm.
