# Supplementary material for: Uniform intratumoral distribution of radioactivity produced using two different radioagents, 64Cu-cyclam-RAFT-c(-RGDfK-)4 and 64Cu-ATSM, improves therapeutic efficacy in a small animal tumor model
Source: EJNMMI Res. 2018 Jun 19;8:54. doi: 10.1186/s13550-018-0407-3 (PMC6008272; doi:10.1186/s13550-018-0407-3)
Supplement: Supplementary file 3 — Intratumoral distribution of 64Cu-ATSM and microvasculature. An adjacent slice to that presented in Fig. 2b was examined by autoradiography, CD31 immunofluorescence staining, and HE staining. Merged image showing 64Cu-ATSM distribution in green, CD31-stained microvessels in red, and HE stains. High-resolution pictures shown by dotted rectangles clearly depicting the stained microvessels in smaller size in 64Cu-ATSM high- vs. low-accumulated areas. Nuclei were stained with DAPI (blue). Scale bars, 2 mm, 200 μm. (PDF 285 kb) [file 13550_2018_407_MOESM3_ESM.pdf]

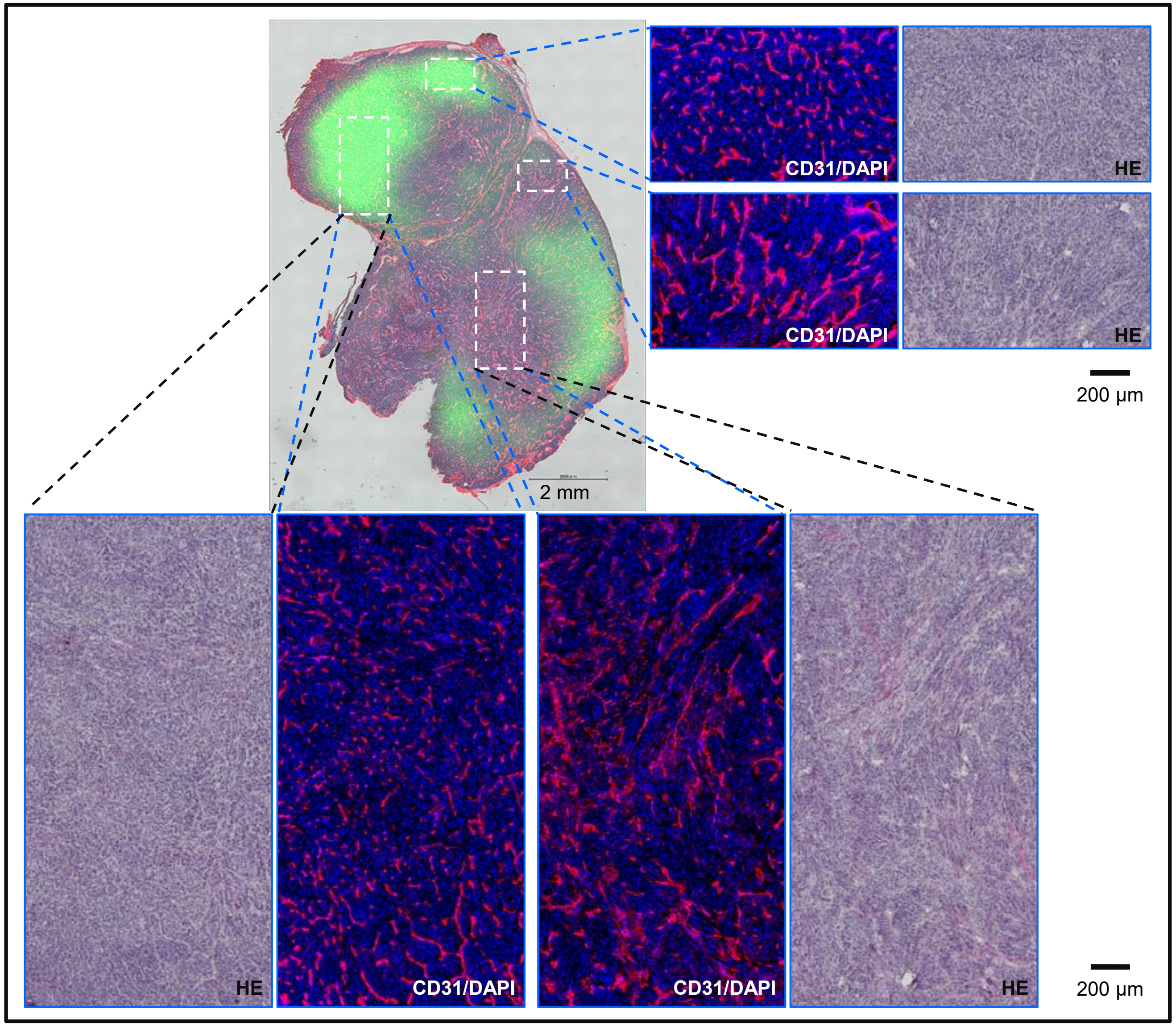


**Additional file 3.** Intratumoral distribution of $^{64}$Cu-ATSM and microvasculature. An adjacent slice to that presented in Figure 2b was examined by autoradiography, CD31 immunofluorescence staining, and HE staining. Merged image showing $^{64}$Cu-ATSM distribution in green, CD31 stained microvessels in red, and HE stains. High resolution pictures shown by dotted rectangles clearly depicting the stained microvessels in smaller size in $^{64}$Cu-ATSM high- versus low-accumulated areas. Nuclei were stained with DAPI (blue). Scale bars, 2 mm, 200 µm.
