# Supplementary material for: Uniform intratumoral distribution of radioactivity produced using two different radioagents, 64Cu-cyclam-RAFT-c(-RGDfK-)4 and 64Cu-ATSM, improves therapeutic efficacy in a small animal tumor model
Source: EJNMMI Res. 2018 Jun 19;8:54. doi: 10.1186/s13550-018-0407-3 (PMC6008272; doi:10.1186/s13550-018-0407-3)
Supplement: Supplementary file 4 — Tumor growth curves of U87MG tumor-bearing mice after single injection of the vehicle solution (control) or 18.5 MBq of 64Cu-RaftRGD (a) or 64Cu-ATSM (b). n = 5–6/group. (PDF 116 kb) [file 13550_2018_407_MOESM4_ESM.pdf]

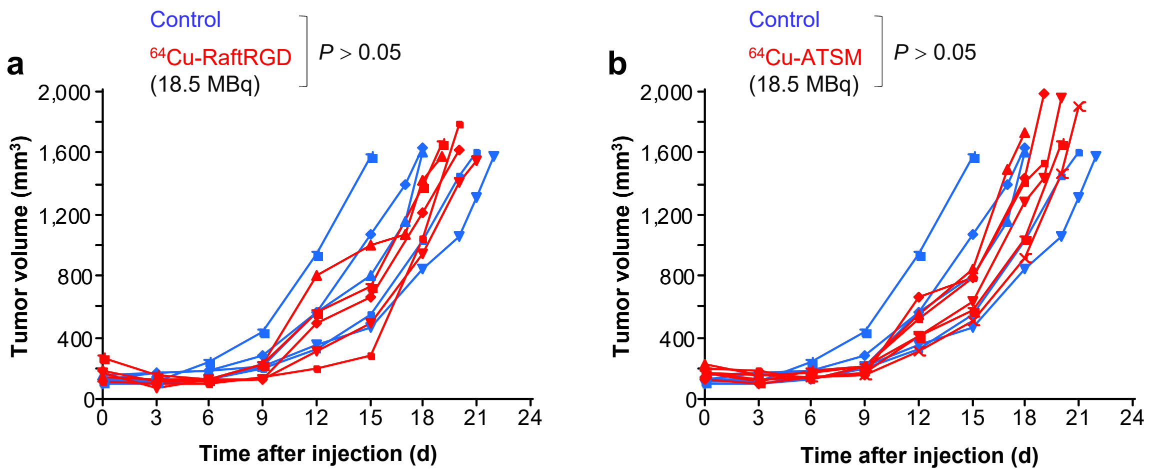

Additional file 4. Tumor growth curves of U87MG tumor-bearing mice after single injection of the vehicle solution (control) or 18.5 MBq of ^{64}Cu-RaftRGD **(a)** or ^{64}Cu-ATSM **(b)**. *n* = 5–6/group.
