# Supplementary material for: Uniform intratumoral distribution of radioactivity produced using two different radioagents, 64Cu-cyclam-RAFT-c(-RGDfK-)4 and 64Cu-ATSM, improves therapeutic efficacy in a small animal tumor model
Source: EJNMMI Res. 2018 Jun 19;8:54. doi: 10.1186/s13550-018-0407-3 (PMC6008272; doi:10.1186/s13550-018-0407-3)
Supplement: Supplementary file 5 — Tumor growth curves (a) and body weight changes (b) of the same set of experimental groups as described in Fig. 3. Values are the means ± standard deviations (n = 6/group). The final data points shown for each group of mice (b) represent the results obtained at survival endpoint days (represented by a mean value). *, †, ‡P < 0.05 for combination, 64Cu-RaftRGD, and 64Cu-ATSM vs. vehicle control, respectively. (PDF 140 kb) [file 13550_2018_407_MOESM5_ESM.pdf]

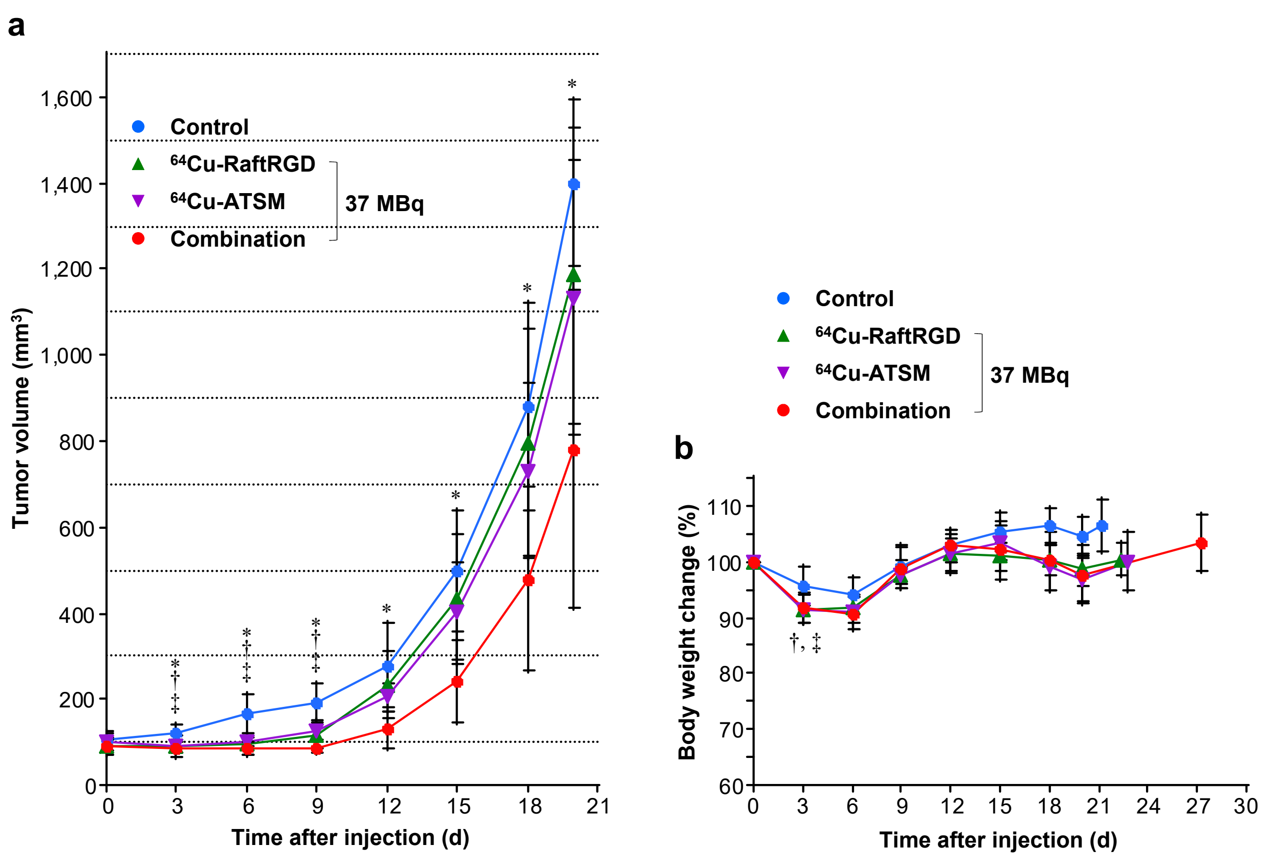

Additional file 5. Tumor growth curves **(a)** and body weight changes **(b)** of the same set of experimental groups as described in Figure 3. Values are the means ± standard deviations (*n* = 6/group). The final data points shown for each group of mice **(b)** represent the results obtained at survival endpoint days (represented by a mean value). $^{*,\dagger,\ddagger}P < 0.05$ for combination, ^{64}Cu-RaftRGD, and ^{64}Cu-ATSM vs. vehicle control, respectively.
