# Supplementary material for: Uniform intratumoral distribution of radioactivity produced using two different radioagents, 64Cu-cyclam-RAFT-c(-RGDfK-)4 and 64Cu-ATSM, improves therapeutic efficacy in a small animal tumor model
Source: EJNMMI Res. 2018 Jun 19;8:54. doi: 10.1186/s13550-018-0407-3 (PMC6008272; doi:10.1186/s13550-018-0407-3)
Supplement: Supplementary file 6 — (a) Tumor growth ratios of the same set of treated groups as described in Fig. 3. *, †, ‡P < 0.05 for combination, 64Cu-RaftRGD, and 64Cu-ATSM vs. vehicle control, respectively. Tumor growth ratios (b) and body weight changes (c) of U87MG tumor-bearing mice after co-administration of 64Cu-RaftRGD and 64Cu-ATSM at 111 MBq (55.5 MBq for each agent) and 148 MBq (74 MBq for each agent). Values are the means ± standard deviations (n = 4/group). *, **P < 0.05 and 0.01, respectively for 111 MBq-group vs. 148 MBq group, respectively. It should be noted that although vehicle controls (b, c) were not performed simultaneously along with the 111 MBq and 148 MBq groups, all the three independent experiments (#1 and #2 extracted from Additional file 4 and Additional file 6a, respectively) showed a reproducibly steady increase of the tumor volume in the vehicle-treated mice. (PDF 337 kb) [file 13550_2018_407_MOESM6_ESM.pdf]

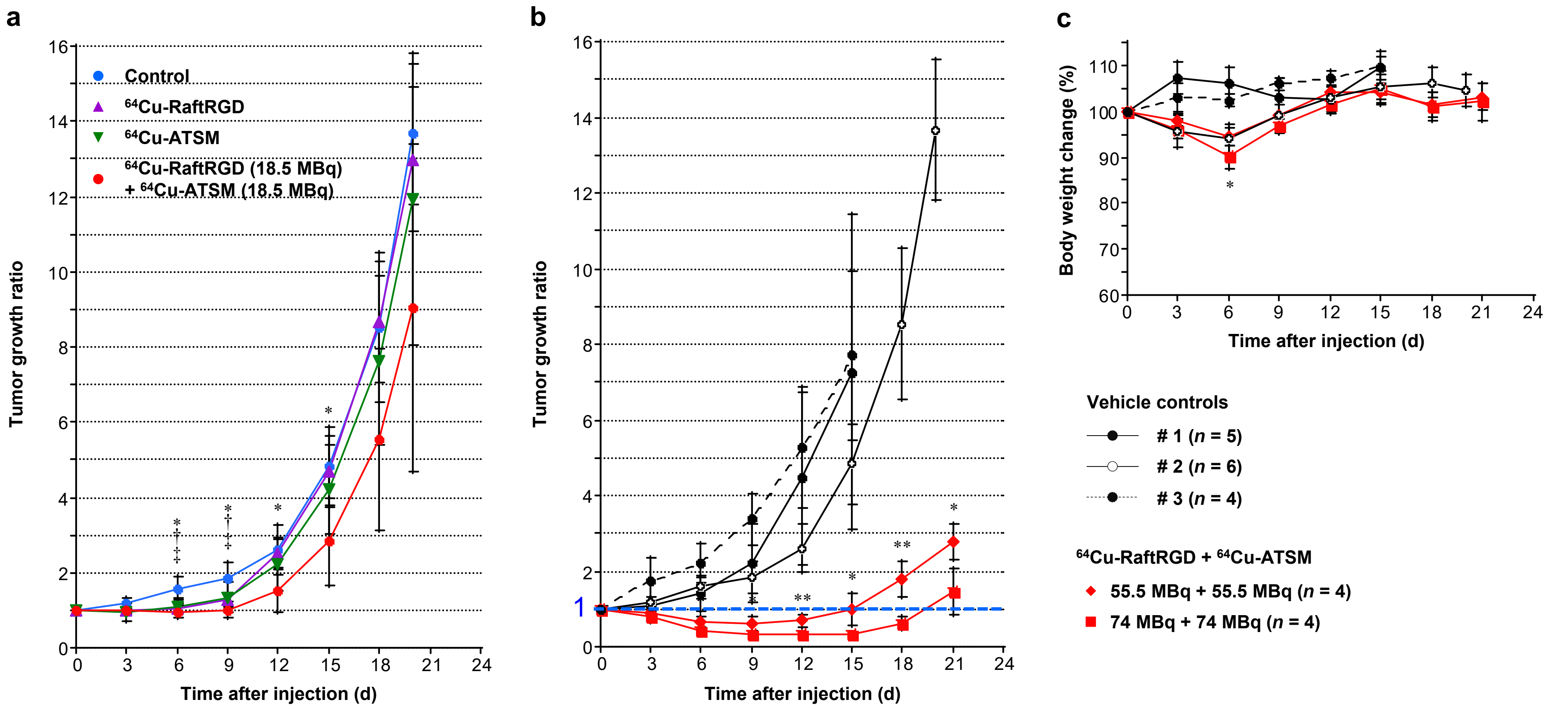

Additional file 6. (a) Tumor growth ratios of the same set of treated groups as described in Figure 3. $^{*,\dagger,\ddagger}P < 0.05$ for combination, ^{64}Cu-RaftRGD, and ^{64}Cu-ATSM vs. vehicle control, respectively. Tumor growth ratios **(b)** and body weight changes **(c)** of U87MG tumor-bearing mice after co-administration of ^{64}Cu-RaftRGD and ^{64}Cu-ATSM at 111 MBq (55.5 MBq for each agent) and 148 MBq (74 MBq for each agent). Values are the means ± standard deviations (*n* = 4/group). $^{*,**}P < 0.05$ and 0.01, respectively for 111 MBq-group vs. 148 MBq-group, respectively. It should be noted that although vehicle controls **(b, c)** were not performed simultaneously along with the 111 MBq- and 148 MBq-groups, all the three independent experiments (# **1** and # **2** extracted from Additional file 4 and Additional file 6 (a), respectively) showed a reproducibly steady increase of the tumor volume in the vehicle-treated mice.
