# Supplementary material for: Uniform intratumoral distribution of radioactivity produced using two different radioagents, 64Cu-cyclam-RAFT-c(-RGDfK-)4 and 64Cu-ATSM, improves therapeutic efficacy in a small animal tumor model
Source: EJNMMI Res. 2018 Jun 19;8:54. doi: 10.1186/s13550-018-0407-3 (PMC6008272; doi:10.1186/s13550-018-0407-3)
Supplement: Supplementary file 7 — Hematology (a) and hepatorenal functions (b) of U87MG tumor-bearing mice after co-administration of 64Cu-RaftRGD and 64Cu-ATSM at 111 or 148 MBq. (PDF 100 kb) [file 13550_2018_407_MOESM7_ESM.pdf]

Additional file 7. Hematology **(a)** and hepatorenal functions **(b)** of U87MG tumor-bearing mice after co-administration of ^{64}Cu-RaftRGD and ^{64}Cu-ATSM at 111 or 148 MBq

a

Group of mice	WBC (10^2/μL)	RBC (10^4/μL)	PLT (10^4/μL)	HGB (g/dL)	HCT (%)	MCV (fL)	MCH (pg)	MCHC (g/dL)
Vehicle[a]	41 ± 9	1042 ± 81	63 ± 7.9	17 ± 1.2	48 ± 3	47 ± 1.7	17 ± 0.3	36 ± 0.7
111 MBq[b]	121 ± 92	1017 ± 36	59 ± 6	18 ± 1.1	49 ± 3.1	48 ± 1.4	18 ± 0.6	36 ± 0.3
148 MBq[b]	71 ± 37	1017 ± 52	58 ± 3.9	18 ± 1	49 ± 2.2	48 ± 0.8	18 ± 0.2	37 ± 0.9

b

Group of mice	Liver enzyme values (U/L)				Renal function indices (mg/dL)	
	GOT	GPT	GGT	ALP	CRE	BUN
Vehicle[a]	123 ± 40	52 ± 15	< 10[c]	322 ± 27	≤ 0.2[c]	19 ± 1
111 MBq[b]	120 ± 17	71 ± 24	< 10[c]	241 ± 36	≤ 0.2[c]	27 ± 3.3
148 MBq[b]	125 ± 31	74 ± 35	< 10[c]	288 ± 25	≤ 0.2[c]	23 ± 3.2

[a]Data are extracted from Supplementary Fig. S2 and Supplementary Table S4 in *Reference 1*, in which normal mice were intravenously injected with the vehicle solution used in the present study, and euthanized on day 20 p.i. for hematology and hepatorenal function tests. It should be noted that same strain of female BALB/cAJcl-*nu/nu* mice were used in both *Reference 1* and the present study.

[b]U87MG tumor-bearing mice (n = 4/each group) were cotreated with ^{64}Cu-RaftRGD and ^{64}Cu-ATSM at 111 MBq (55.5 MBq for each agent) and 148 MBq (74 MBq for each agent), and euthanized on day 21 p.i. for hematology and hepatorenal function tests.

[c]All the values are below or equal to the lower limit of measurement.

The data are presented as the means ± standard deviations. There were no significant differences in all these parameters between the 111 MBq and 148 MBq groups.

Reference 1

Jin ZH, Furukawa T, Degardin M, et al. $\alpha_V\beta_3$ Integrin-targeted radionuclide therapy with ^{64}Cu-cyclam-RAFT-c(-RGDfK-)$_4$. Mol Cancer Ther. 2016;15(9):2076–85.
